# Characterization of the Effects of Ligands on Bonding and σ-Aromaticity of Small Pt Nanoclusters

**DOI:** 10.1021/acs.jpca.2c08614

**Published:** 2023-05-08

**Authors:** Samantha Reid, Heriberto Hernández

**Affiliations:** Department of Chemistry, Grinnell College, Grinnell, Iowa 50112, United States

## Abstract

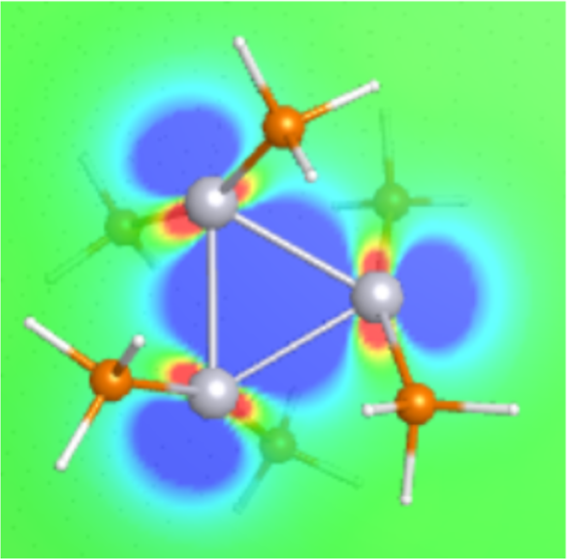

Nanoclusters, particularly gold nanoclusters, have attracted the attention of researchers due to their potential applications in the medicine and energy fields. Other noble-metal nanoclusters, including Pt, have also been studied, but in lesser detail. Pt is known for its excellent catalytic properties and is a promising candidate for applications in catalysis and biomedicine. In this study, we used density functional theory to elucidate the molecular and electronic structures of small phosphine-ligated Pt nanoclusters. This study is directed at identifying highly stable platinum clusters. Our results show that phosphine-ligated platinum nanoclusters with σ-aromaticity have high stability. In addition, we were able to predict the most stable clusters using an electron counting equation.

## Introduction

A nanocluster is a group of a few atoms forming a particle smaller than 2 nm,^1^ while nanoparticles are larger clusters of atoms up to 10 nm in size.^2^ Noble metal nanoclusters have been the subject of study in the last two decades,^3,4^ mainly because of their unique properties that originate from their size and shape. Owing to its potential applications in catalysis, gold is one of the most studied noble metal nanoclusters.^1,3,5,6^ Copper, silver, and Pt nanoclusters have also been the subject of study in recent years.^1,7−11^ Pt is traditionally known for its catalytic activity^12^ and its use in therapeutic cancer drugs.^2^ However, in general, nanoclusters can affect a range of fields, including biomedicine, solar energy conversion, nanoscale electronics, optical imaging, and chemical sensing.

Computational studies have examined the molecular and electronic structures of bare Pt nanoclusters.^13−15^ Another study examined the molecular structure and vibrational features of platinum–phosphorus mixed clusters, Pt_*n*_P_2*n*_ (*n* = 1–5), using density functional theory (DFT).^16^ Meanwhile, Huang and Lee used a cluster model approach to simulate the adsorption of CO on a Pt surface.^13^ The study concluded that the size of the cluster impacts the adsorption of CO. It is well known that the size of a particle plays an important role in its electronic properties and is the basis for examining ultrasmall Pt nanoclusters in this study. Another study by Aprà and Fortunelli concluded that Pt_13_–Pt_55_ exhibited metallic characteristics, which also prompted us to examine Pt nanoclusters with a small number of Pt atoms. Most computational studies focus their attention on bare clusters; however, it is important to note the important role that ligands play in the solution-based synthesis of nanoclusters.^17^ Ligands are also essential in controlling the size and distribution of nanoclusters.^18^ Approximately 38 years ago, an experimental study by Klevtsova et al. reported on the crystal structure of a carbonyl and phosphine-ligated Pt_4_L_8_ nanocluster.^19^ In Klevtsova’s study, the X-ray crystallography data revealed a tetrahedral Pt core structure with Pt–Pt distances ranging from 2.69 to 2.77 Å. A second study by Hendrickson et al. used ion cyclotron resonance mass spectrometry with electron impact ionization.^20^ The molecular ion found, Pt_4_(PF_3_)_8_^+^, was a byproduct of chemical vapor deposition of the Pt(PF_3_)_4_ compound. Both studies provide experimental evidence of the formation of a stable phosphorous-ligated Pt nanocluster.

Furthermore, computational studies that focus on ligand-base Pt nanoclusters are limited. A theoretical study by Evans^21^ examined the electronic and structural features of Pt phosphine hydride clusters using the Extended Hückel molecular orbital theory. They reported that Pt_4_L_8_ (L = ligand) nanoclusters have a tetrahedral structure. Evans’s theoretical results produced the same molecular structure as the experimental results reported by Klevtsova. These results are also evidence of the predictive power of theoretical chemistry.

In this study, we explored the molecular and electronic structures of phosphine-ligated Pt nanoclusters using DFT, time-dependent DFT, and natural bond orbital analysis (NBO). The clusters studied have 2–5 Pt atoms, with phosphine (PH_3_) and trimethylphosphine (PMe_3_) as ligands. For the first time, we report on the theoretical evidence of σ-aromaticity in phosphine and trimethylphosphine-ligated Pt nanoclusters.

## Theoretical and Computational Details

We performed ab initio computations on Pt_*n*_(PH_3_)_2*n*_ and Pt_*n*_(PMe_3_)_*m*_ with *n* = 2–5 and *m* = 2–8 and their analogous charged clusters using the Gaussian suite of programs.^22^ All the structures were optimized using DFT. We used two functionals, the B3LYP^23,24^ and the PW91,^25^ in combination with the def2TZV^26^ triple zeta basis set. We also computed the vibrational frequencies of all the sample structures and confirmed that every cluster was a local minimum. Additionally, we performed an electronic structure analysis using the NBO,^27^ where we examined the NBO charges, HOMO–LUMO gap, and NBO bonds formed between Pt atoms. We calculated the number of electrons available for bonding in the cluster (*n*_e_) using eq 1
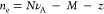
1where *N* is the number of metal atoms, ν_A_ is the atomic valency, *M* is the number of electron-withdrawing ligands, and *z* is the overall charge.

### Method Validation

To validate our level of theory, we performed ab initio computations on the neutral bare platinum 2 clusters and the charged and neutral phosphine-ligated platinum 2 clusters. We used the coupled cluster, CCSD,^28^ in combination with the def2TZV triple zeta basis set and compared the results with three different functionals. Two functionals, PBE^25^ and PW91, have been previously used on bare platinum clusters.^29,30^ In contrast, the PBE and B3LYP have been previously used in trimethyl phosphine^31^ and phosphine-ligated^18^ platinum and gold clusters, respectively. Results for the Pt–Pt and Pt–P bond distances, in Å, are shown in Table 1.

**Table 1:** Bond Distances in Angstroms for the Optimized Structures of the Neutral Bare Pt_2_ Cluster and the Neutral and Charged Phosphine-Ligated Platinum Clusters at Various Levels of Theory

		bond distances in angstroms			
cluster	bond	CCSD/def2TZV	B3LYP/def2TZV	PBE/def2TZV	PW91/def2TZV
Pt_2_	Pt–Pt	2.403	2.378	2.378	2.377
Pt_2_(PH_3_)_4_	Pt–Pt	2.917	2.993	2.808	2.849
	Pt–P	2.321	2.319	2.230	2.301
Pt_2_(PH_3_)_4_^2+^	Pt–Pt	2.535	2.570	2.550	2.549
	Pt–P	2.403	2.400	2.382	2.382

These results show that for the bare Pt_2_ cluster, all three functionals’ performance is identical and is within a 1% difference of the CCSD results. These results are also consistent with previous theoretical results of 2.37 Å for the Pt–Pt bond distance in the Pt_2_ cluster.^32^ When examining the neutral ligated clusters, the PBE results show the most significant difference (4%) for the platinum–phosphorus interaction, while the B3LYP (0.1%) and the PW91 (0.9%) are closer to the coupled cluster results. Similarly, for the platinum–platinum interaction, the PBE has the most considerable difference (3.7%). The only time the PBE functional performance is less than 1% is for the charged cluster.

Nevertheless, the PW91 has identical results for the charged clusters as PBE. The B3LYP has the closest value compared to the coupled cluster results for the Pt–P. For the Pt–Pt bond, the B3LYP is within a 1.4% difference. Because bonding interactions are a fundamental part of this research, all the structures are computed using the B3LYP/def2TZV and the PW91/def2TZV levels of theory. In addition to this level of theory, we included the Douglas–Kroll–Hess second-order scalar relativistic Hamiltonian, dkh2,^33,34^ in all our energy computations, including time-dependent DFT (TD-DFT) calculations and NBO analysis. The results and discussion show the values of distances and energies for the B3LYP functional, with the PW91 results in parenthesis. Note: The HOMO–LUMO energy gaps (*E*_gap_) were taken from the TD-DFT analysis at the mentioned levels of theory rather than using the output energies from the molecular orbitals.

## Results and Discussion

Small Pt nanoclusters containing two to five atoms and various ligands were optimized using DFT. For clusters containing 4 and 5 Pt atoms, we used tetrahedral and bipyramidal structures as starting geometries, respectively. As stated above ,^19^ X-ray crystallography data revealed a tetrahedral Pt core structure for the Pt_4_(CO)_5_(Pet_3_)_4_ cluster. For Pt_5_, previous theoretical results showed that a bipyramidal structure is adopted when ligands are present.^21^

**Figure 1:**
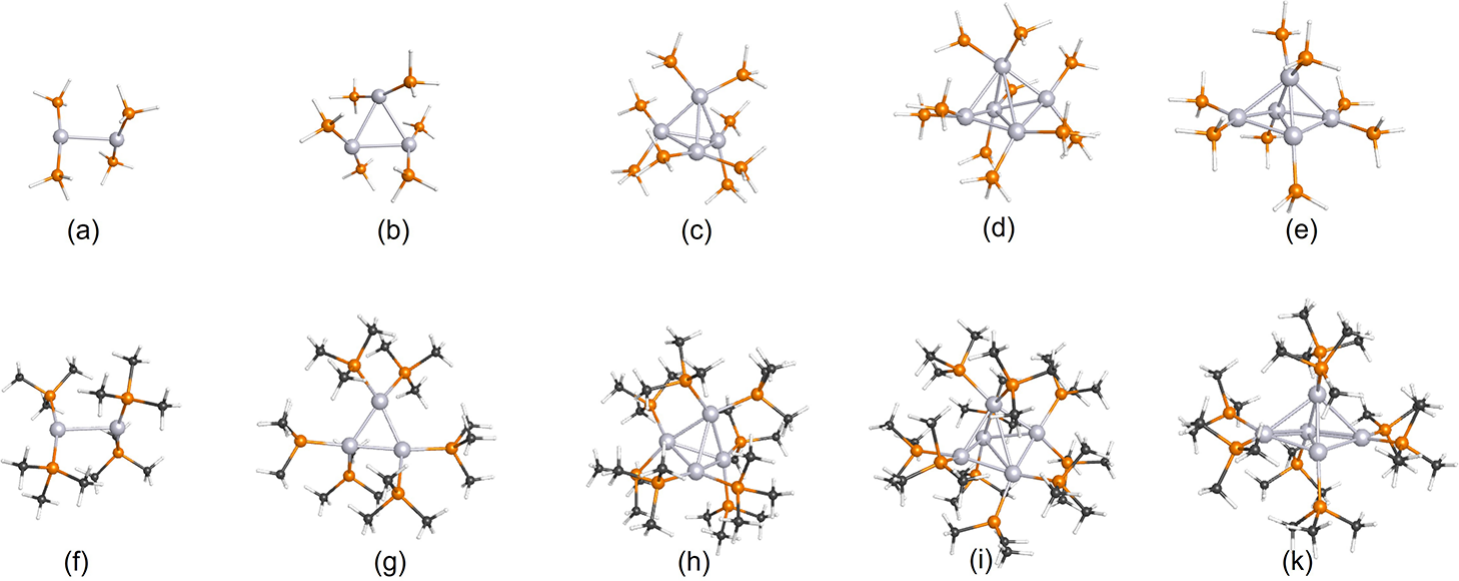
Optimized structures of Pt_*n*_(PH_3_)_2*n*_ (*n* = 2–5) and Pt_*n*_(PCH_3_)_2*n*_ (*n* = 2–5). Optimizations were performed using the B3LYP/def2TZV and PW91/def2TZV levels of theory. Color code: hydrogen—white, carbon—black, phosphorus—orange, and platinum—gray.

The two main ligands used in this study were phosphine and trimethylphosphine, with a platinum-to-ligand ratio of 2, except for Pt_5_(PCH_3_)_8_. The lower number of ligands in the former cluster is due to the geometric constraints, which we attributed to the steric hindrance between the ligands. Figure 1a,f shows the Pt_2_(PH_3_)_4_ and Pt_2_(PCH_3_)_4_ nanoclusters, respectively. The Pt–Pt distances were 2.993 (PW91, 2.851) and 3.053 (PW91, 2.886) Å, respectively.

Figure 1b,g shows the optimized structures for the Pt_3_(PH_3_)_6_ cluster and the Pt_3_(PMe_3_)_6_ cluster, respectively. Both clusters are highly symmetrical, whereas the phosphine-ligated cluster is slightly more symmetrical, featuring equal bond lengths between every Pt atom. Figure 1c,h illustrates the optimized clusters for Pt_4_(PH_3_)_8_ and Pt_4_(PMe_3_)_8_, respectively. These clusters have a tetrahedral core structure. Finally, Figure 1d,i shows the optimized structures for Pt_5_(PH_3_)_10_ and Pt_5_(PMe_3_)_10_. Figure 1e,k shows the optimized structures for Pt_5_(PH_3_)_8_ and Pt_5_(PMe_3_)_8_. We used eight ligands rather than 10 to minimize the steric hindrance in the ligands. This change results in a more symmetric cluster. For example, the Pt_5_(PMe_3_)_10_ base-atoms (lower four atoms in Figure 1k) have Pt–Pt bond distances ranging from 2.65 to 2.99 Å. On the contrary, the Pt_5_(PMe_3_)_8_ base-atoms (lower four atoms in Figure 1i) have equal Pt–Pt bond lengths of 2.67 Å. We also performed a ligand dissociation-energy (*D*_e_) calculation, and both Pt_5_L_8_ (L = PH_3_, PMe_3_) clusters have higher *D*_e_ values (9.89 and 10.69 eV, respectively) when compared to the Pt_5_L_10_ (L = PH_3_, PMe_3_) clusters (9.45 and 9.94 eV, respectively). In addition, PMe_3_ has a higher dissociation energy than PH_3_ in both cases. This trend is consistent with phosphine-ligated Au clusters, where the strength goes from PH_3_ to PMe_3_ to PPhe_3_. Furthermore, Nair et al. found experimental evidence of a stable triphenylphosphine-ligated platinum cluster, Pt_17_(CO)_12_(PPh_3_)_8_.^35^

### Binuclear-Platinum Cluster

**Figure 2:**
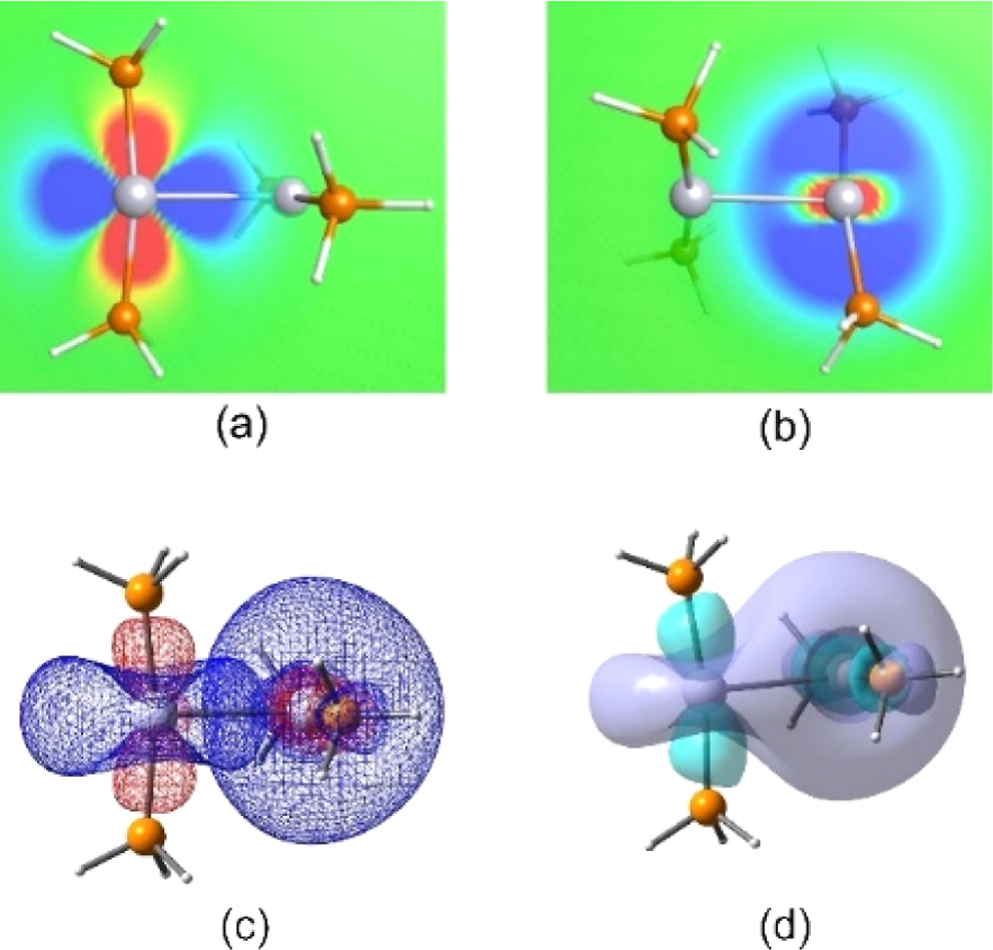
Optimized structure of the neutral Pt_2_(PH_3_)_4_ cluster. (a) Platinum lone pair (*n*), which donates electron density to (b) the Lewis valence orbital of the adjacent platinum; in (c), we show the interaction of both orbitals leading to the *n*_(Pt)_ → LV_(Pt)_ interaction. In (d) is the isosurface for the addition of the cubes of the orbitals shown in (c). The isovalue used for the surface contours is 0.04. Color code: Platinum—silver, phosphorus—orange, and hydrogen—white.

The binuclear cluster Pt_2_(PH_3_)_4,_ is the simplest of all the structures. From the optimized structures, it was evident that both ligands, phosphine and trimethylphosphine, were turned in opposite directions because of the steric hindrance of the ligands to form the Pt–Pt bond. The Pt_2_L_4_^2+^ cluster had two charges because the neutral cluster cannot form a strong Pt–Pt bond. This indicates that a neutral cluster does not exist, while a charged cluster does. According to our calculations, there are four electrons available for bonding (*n*_e_) between the Pt atoms in the Pt_2_(PH_3_)_4_ cluster, as predicted using eq 1. In principle, a large electron density around both the Pt atoms yields a type of covalent bonding upon interaction, rather than ionic bonding. To describe the bonding between the Pt atoms, we performed a NBO analysis on all optimized structures. We found a strong interaction between one of the lone pair orbitals of Pt, *n*_Pt(1)_, and the Lewis valence (LV) orbital of the opposite Pt atom, LV_Pt(2)_. These orbitals are shown in Figure 2a,b. The overlap of these orbitals produces *n*_Pt(1)_ → LV_Pt(2)_ interactions. Further analysis showed that the population on the LV orbital of Pt(1) was 0.64 electrons (PW91, 0.69 electrons).

**Figure 3:**
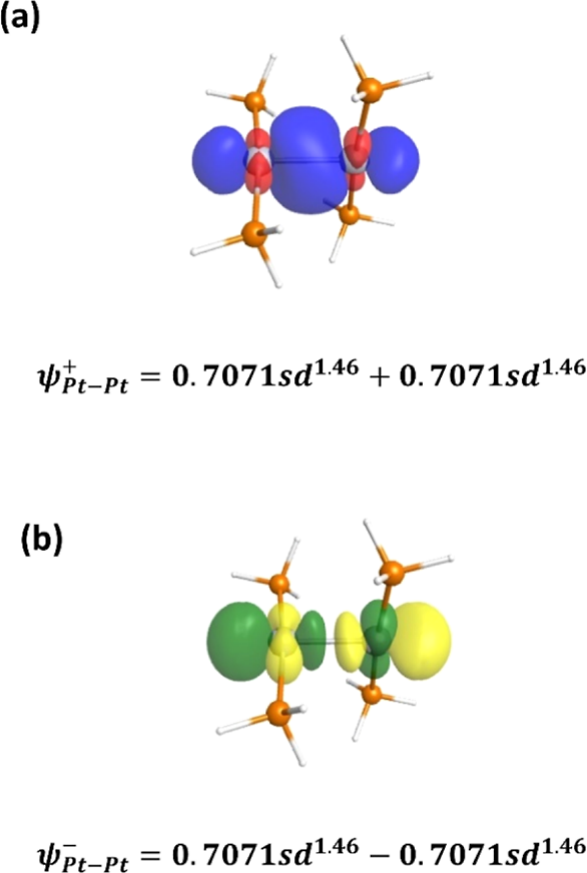
Visual representation of (a) the Pt–Pt NBO sigma-type bonding orbital, σ_Pt–Pt_, with a bond distance of 2.570 Å (PW91, 2.549 Å) and (b) the Pt–Pt NBO sigma-type anti-bonding orbital. Underneath each figure is the wavefunction that describes the bonding (ψ^+^) and anti-bonding (ψ^–^) orbitals in the charged Pt (2) cluster. A similar bonding is also present in the Pt_2_(PMe_3_)_4_^2+^ nanocluster, Figure S1, with a Pt–Pt bond distance of 2.576 Å (PW91, 2.550 Å).

Similarly, the Pt atom receiving the electron density, Pt(2), had the same electronic structure as the donating Pt atom, Pt(1). Therefore, the same type of interaction can occur in reverse, resulting in *n*_(Pt2)_ → LV_(Pt1)_ interactions. This interaction also yields a 0.6 electron density in the Lewis valence orbital of Pt(1). The overlap of these orbitals, as shown in Figure 2c, yields a strong interaction. Figure 2d shows the addition of a lone pair and Lewis’s valence-orbital cubes. This type of interaction increased the number of weak covalent bonds, where one electron is shared between two Pt atoms. Since there were four (4) electrons available for bonding, we expected that a lower electron density would result in less steric hindrance and a better overlap between the lone pair and Lewis’s valence-orbitals. Therefore, we examined the effect of electron-donating and electron-withdrawing ligands on the binding between the Pt atoms. First, we evaluated the binuclear Pt cluster with trimethylphosphine ligands. Upon comparison of the Pt–Pt distances, we found that the trimethylphosphine-liganded cluster had a slightly larger bond distance, 3.053 Å (PW91, 2.886 Å) compared to that of the phosphine ligand, 2.993 Å (PW91, 2.851 Å). To investigate our hypothesis further, we optimized the neutral binuclear cluster using trifluorophosphine. We hypothesized that by using the electron-withdrawing group PF_3_, we can demonstrate that reducing the electron density between the Pt atoms will lead to a stronger interaction and therefore a shorter bond distance. The results for the optimized structure of Pt_2_(PF_3_)_4_ indicated a bond length of 2.801 Å (PW91, 2.702 Å) between the Pt atoms. This not only confirms the hypothesis but also provides insight into the type of cluster that is more stable. Based on these results, we optimized the analogous charged clusters Pt_2_(PH_3_)_4_^2+^ and Pt_2_(PCH_3_)_4_^2+^. We foresaw that by removing two electrons from the cluster, we could obtain a stronger interaction between the Pt atoms compared with having neutral ones. Our findings reveal that the charged clusters indeed have a shorter bond distance between the Pt atoms than their neutral counterparts. Not only are the distances shorter but the NBO analysis results indicate a sigma bond between the Pt atoms, as shown in Figure 3.

From the first perspective, the charged cluster appears to be more stable than its neutral counterpart. We used the HOMO–LUMO gap (*E*_gap_) as the criterion for assessing stability. We found that the charged cluster had a lower *E*_gap_ of 1.22 eV (PW91, 1.11 eV) than that of the neutral cluster, 2.62 eV (PW91, 2.16 eV). The smaller *E*_gap_ indicates that the charged cluster is more reactive than the neutral cluster. Further analyses were performed using TD-DFT at the B3LYP/def2TZV dkh2 and PW91/def2TZV dkh2. The resulting UV/vis data show that the charged cluster had a larger wavelength for maximum absorption, λ_max_ = 1029.4 nm (PW91, 1112.0 nm), and the neutral cluster had a shorter wavelength at λ_max_ = 473.2 nm (PW91, 574.4 nm). Nevertheless, the fact that the charged cluster is more reactive does not mean that the bonding interaction between the Pt atoms is weaker than that of its neutral counterpart.

### Clusters Containing More than Two (2) Pt Atoms

The results of the NBO analysis show that both neutral and charged clusters have sigma-type bonding. There is also a correlation between the number of sigma bonds and the number of electrons available for bonding *n*_e_. As shown in Table 2, the neutral Pt_3_(PH_3_)_6_ cluster has six electrons available for bonding and forms three sigma bonds, whereas the charged cluster has only four electrons available and consequently forms two bonds. As presented on the right side of the orbital column, Pt_3_(PH_3_)_6_ had a molecular orbital that was delocalized between the three Pt atoms. Meanwhile, the charged cluster, Pt_3_(PH_3_)_6_^2+^, has a molecular orbital connecting the three atoms but is not delocalized as in the neutral cluster. Finally, the last column of Table 2 shows the HOMO–LUMO energy gaps (*E*_gap_). The neutral Pt_3_ cluster had a slightly larger *E*_gap_ than its analogous charged cluster. As previously mentioned, a larger *E*_gap_ indicates stability, which is consistent with the type of resonance found in the neutral cluster. This delocalization is a type of aromaticity found in small metal nanoclusters.^36−38^ These theoretical works discussed aromaticity in transition metal systems consisting of bare clusters with no ligands. In contrast, the developed system consists of phosphine-ligated clusters. The results from molecular orbital theory calculations show that neutral clusters containing three to five atoms exhibited σ-aromaticity. This is consistent with the type of aromaticity described in Zubarev’s paper.^38^ For Pt_3_, the electron density was seen to be delocalized between the three Pt atoms. The origin of this interaction can be attributed to the overlap of the d_*z*_^2^ orbitals of Pt interacting head-to-head. The six electrons are properly delocalized among the three atoms, giving the cluster higher stability compared to that of the three charged Pt clusters. In addition, the *E*_gap_ for the neutral Pt_3_ cluster was larger than that of the charged cluster. As shown in Table 2, the Pt_4_ cluster has a spherical electron density owing to its tetrahedral geometry, with eight electrons completely delocalized in the center of the cluster. *E*_gap_ is also larger for the neutral cluster compared to that of its counterpart. In addition, the *E*_gap_ difference between the neutral and charged clusters was larger than that in the previous clusters. The last cluster, Pt_5_, also exhibited σ-aromaticity. In this case, the bipyramidal structure shows a diamond-shaped electron density at the center of the cluster, which is consistent with the σ-aromaticity. The *E*_gap_ value for the latest neutral cluster, Pt_5_, was also greater than that of the charged cluster. There were several significant findings in this study. First, the neutral clusters containing 2–4 Pt atoms have energy gaps larger than 2 eV (PW91, 1.95 eV), indicating stability. Second, the charged Pt_3_ cluster has an *E*_gap_ of 1.79 eV (PW91,1.51 eV), which is larger than all the charged clusters, implying that it is indeed a stable cluster and therefore a possible experimental candidate. Additionally, we observed the same type of σ-aromaticity in the neutral trimethyl phosphine-ligated clusters, as shown in Figure S2.

**Table 2:**
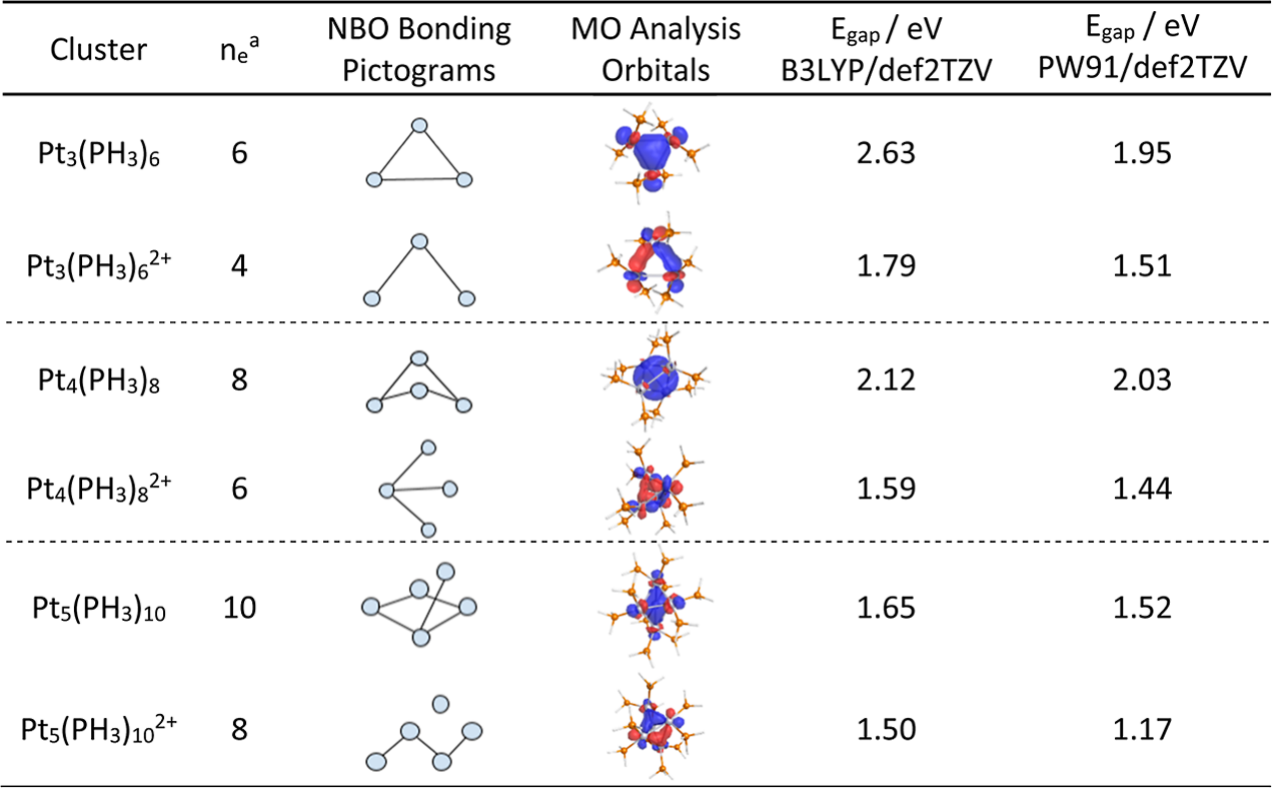
Neutral and Charged Nanoclusters Containing Three to Five Pt Atoms with the Number of Electrons Available for Bonding (*n*_e_), the Number of Sigma Type Bonds According to NBO Analysis (Pictogram), and the Molecular Orbital Containing the Pt Atoms and the HOMO–LUMO Energy Gap

a *n*_e_ is the number of electrons available for bonding (eq 1).

**Figure 4:**
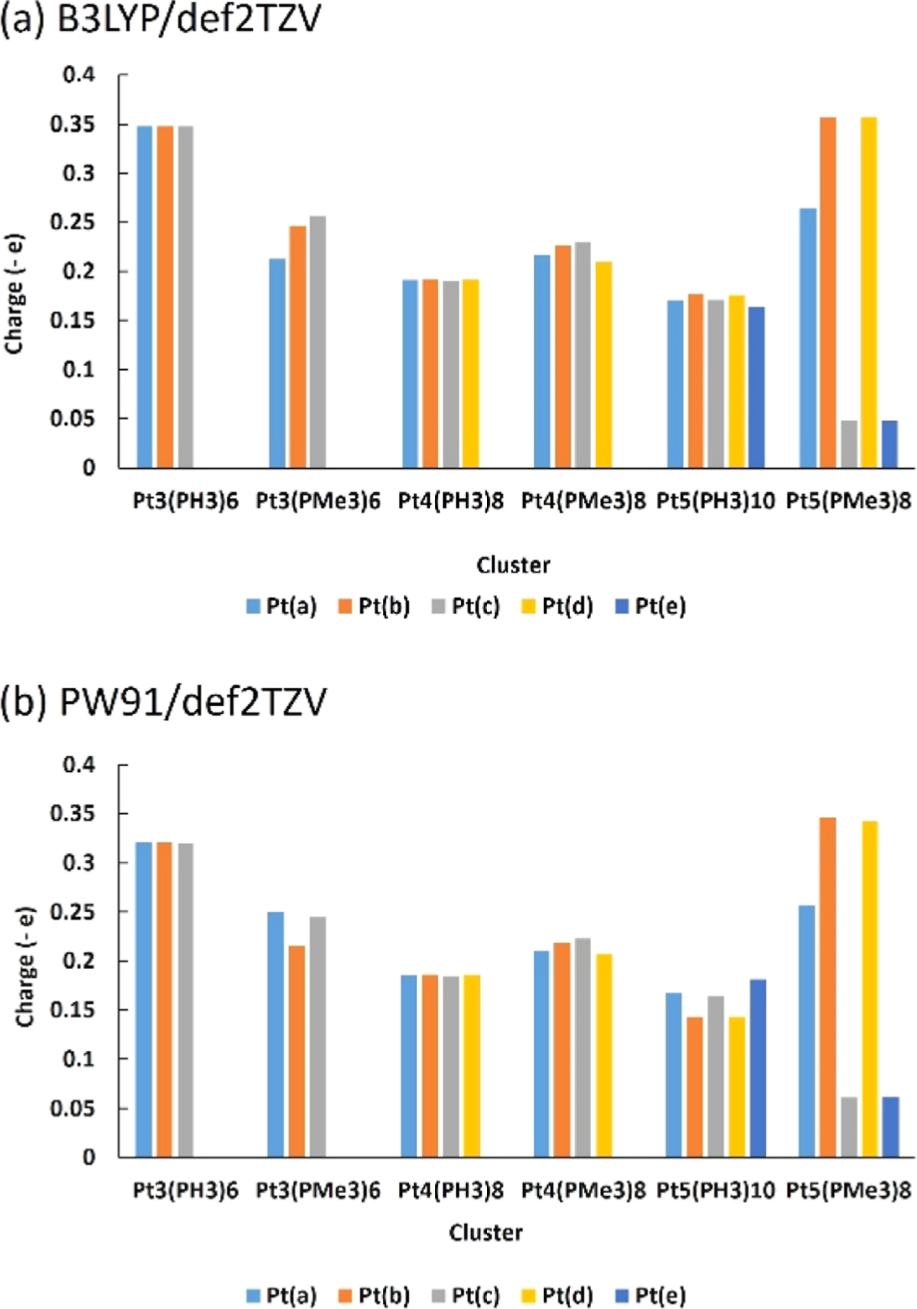
Graph showing the NBO charges of the Pt atoms for each optimized structure at (a) the B3LYP/def2TZV dkh2 and (b) PW91/def2TZV dkh2 levels of theory, where Pt(a), Pt(b), and so on refer to the label of each atom that forms a cluster. Note: All the charges are negative and were taken into account in the units (−*e*).

We also examined the NBO charges to aid in understanding the findings regarding σ-aromaticity. Figure 4 shows the NBO charges for all neutral clusters containing both phosphine (PH_3_) and trimethylphosphine (PMe_3_) ligands. For the Pt_3_ and Pt_4_ structures, the clusters with the trimethylphosphine ligand had slightly fewer equal charges than the partnering cluster with the phosphine ligands. In the case of the Pt_3_ cluster, the phosphine-ligated cluster had exactly equal charges on each Pt atom, as determined by natural population analysis (NPA). All the P atoms in this cluster had equal charges, which likely led to the core atoms having equal charges as well. By examining the NPA of the trimethylphosphine-ligated Pt_3_ cluster, it can be seen that the core Pt atoms do not have exactly equal charges. As shown in Figure 1g, the methyl groups adopt different positions depending on their interactions with other groups. Therefore, this inequality in charge is due to the methyl groups, which led to P not having all equal charges, thereby slightly changing the charge of each core Pt atom. The same pattern of charges was observed in the NPA for the phosphine-ligated and trimethylphosphine-ligated Pt_4_ structures. This result indicates that the charges of the Pt atoms at the core of the cluster were influenced by the ligands; therefore, the stability of the cluster is influenced by the ligands.

An obvious example of this charge pattern for Pt_5_ clusters is shown in Figure 4. For the phosphine-ligated structure, the charges were more equally distributed, as in the previous phosphine-ligated clusters. In contrast, the five-platinum structure with eight ligands, Pt_5_(PMe_3_)_8_ had the least stable structure because it had the most variable charges for the Pt atoms. We were able to optimize the Pt_5_(PMe_3_)_8_ cluster with eight ligands because the methyl groups occupied more space, and therefore attaching 10 ligands was impossible. The steric effects of the methyl groups were more evident in this case than in the previous clusters because of the large number of groups. Finally, we examined the charges of the Pt_2_L_2_ and Pt_2_L_2_^2+^ (L = PH_3_, PMe_3_) clusters, shown in Figure S3. The NPA results indicate that the neutral cluster had negative charges on both Pt atoms. This result is consistent with that for neutral clusters, as stated above and shown in Figure 4. Meanwhile, Pt_2_(PH_3_)_4_^2+^ had positive charges on both Pt atoms, while Pt_2_(PMe_3_)_4_^2+^ had one positive Pt atom and the other was negative.

We also performed TD-DFT computations to calculate the wavelength of the maximum absorption λ_max_. The values are tabulated in Table 3, which lists the λ_max_ for phosphine-ligated clusters on the left side and trimethylphosphine-ligated clusters on the right side. As seen from these data, the typical pattern for all clusters, aside from Pt_2_L_4_^2+^, is that λ_max_ for the trimethylphosphine-ligated clusters is larger than that for the phosphine-ligated clusters. According to our results, the Pt_2_ clusters follow an opposite trend, as shown in Table 3. From this table, it can be seen that the trimethylphosphine-ligated Pt_2_L_4_ cluster has a smaller λ_max_ than the phosphine-ligated cluster. This result was expected because the PMe_3_ ligand is known to be an electron-withdrawing group, and as shown above, it stabilizes the bonding between the Pt atoms. Therefore, it was expected that the stability of the trimethylphosphine-ligated cluster would be greater than that of the phosphine-ligated cluster.

**Table 3:** TD-DFT Results of the Wavelength of Maximum Absorption for the Neutral Phosphine- and Trimethylphosphine-Ligated Pt Nanoclusters Computed Using the B3LYP and the PW91 Methods in Combination with the Douglas–Kroll–Hess Second-Order Scalar Relativistic Hamiltonian, dkh2, and the Triple Zeta, def2TZV, Basis Set

	B3LYP/def2TZV dkh2		PW91/def2TZV dkh2	
cluster	λ_max_, L = PH_3_	λ_max_, L = PMe_3_	λ_max_, L = PH_3_	λ_max_, L = PMe_3_
Pt_2_L_4_	473.9	441.4	574.4	506.3
Pt_3_L_6_	469.1	618.1	570.8	771.8
Pt_4_L_8_	584.8	589.2	608.5	530.2
Pt_5_L_10_	602.8	619.8	896.7	693.1
Pt_5_L_8_	687.4	762.9	748.6	779.5

We analyzed the transitions from the TD-DFT calculations for the neutral Pt clusters to explain the resultant λ_max_ of absorption and its corresponding orbital transitions. Our results show that Pt_2_(PH_3_)_4_ has a λ_max_ of 574.4 nm, which corresponds to the transition from molecular orbital 54 (HOMO) to molecular orbital 55 (LUMO) and molecular orbital 54 (HOMO) to molecular orbital 57 (LUMO + 1), see Figure S5. These transitions correspond to an *E*_gap_ of 2.16 eV at the PW91/def2TZV dkh2 level of theory. A second absorption peak is also present in the spectra of Figure S2 with a wavelength of 540 nm (2.29 eV). The origin of this peak in the spectrum is due to a transition from the HOMO to the LUMO + 2 molecular orbitals [MO(54) → MO(57)]. Other clusters with λ_max_ corresponding to the HOMO → LUMO transition are those containing PMe_3_ as a ligand, Pt_2_(PMe_3_)_4_, Pt_3_(PMe_3_)_6_, Pt_5_(PMe_3_)_8_, and Pt_5_(PMe_3_)_10_, except for Pt_4_(PMe_3_)_8_.

**Figure 5:**
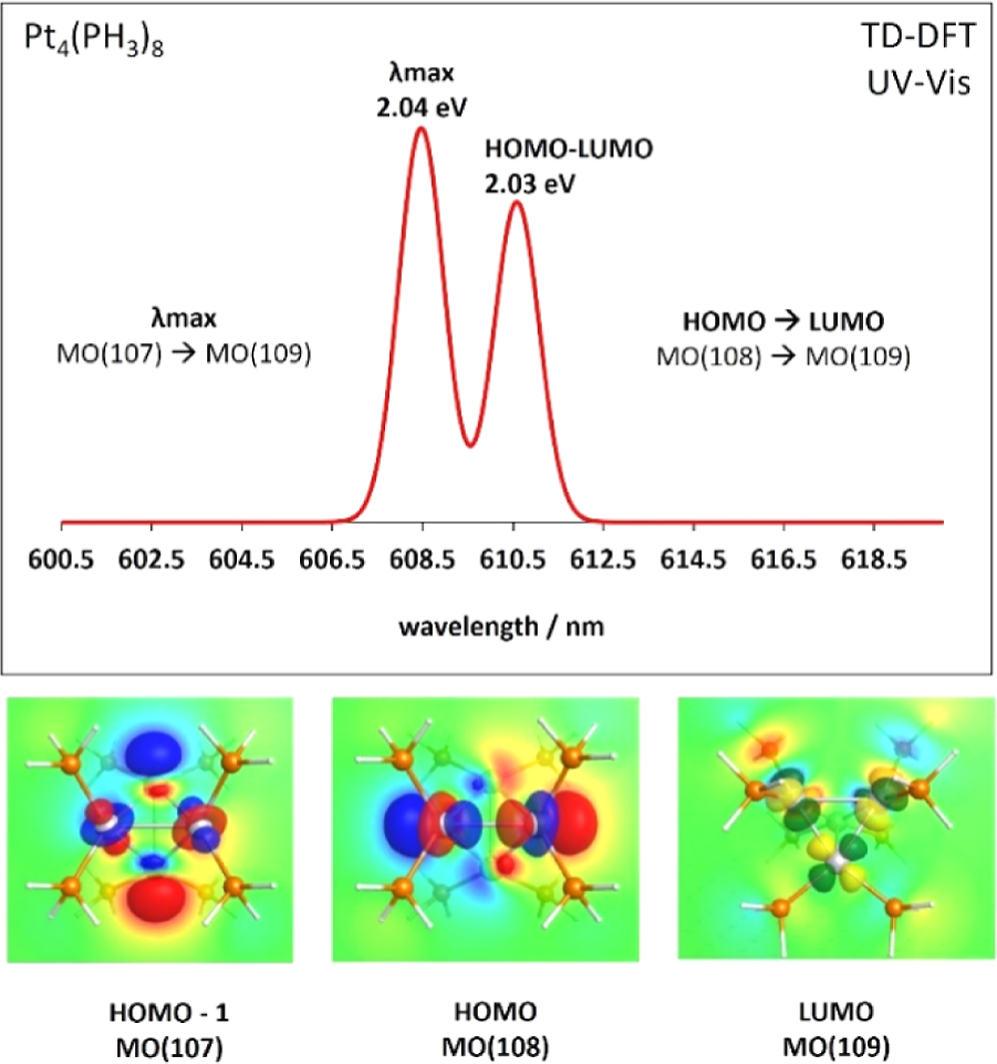
UV–vis spectrum of the neutral Pt_4_(PH_3_)_8_ cluster and its corresponding transitions resulting in the absorption spectrum. Underneath the spectrum are the plots of the molecular orbitals involved in the transitions. All the data were obtained using TD-DFT at the PW91/def2TZV dkh2 level of theory. Note: We used a slicing plane in all three orbitals’ plots in order to help with visualization.

For Pt_3_(PH_3_)_6_, Pt_4_(PH_3_)_8_, and Pt_5_(PH_3_)_8_ clusters, the maximum absorption spectra correspond to the transition from the HOMO – 1 to the LUMO, rather than the HOMO → LUMO electronic transition. The HOMO → LUMO transition is present but not as the maximum absorption. An example of this is shown in Figure 5 for the Pt_4_(PH_3_)_8_ cluster. As in the previous example, the TD-DFT level of theory is PW91/def2TZV dkh2. In the spectrum, the HOMO → LUMO transition [MO(108) → MO(109)] has an absorption wavelength of 610.5 nm (2.03 eV). While the wavelength of maximum absorption, λ_max_, corresponds to a transition from the HOMO – 1 to the LUMO [MO(107) → MO(109)]. Underneath the spectrum are the electron densities of the corresponding molecular orbitals. A plane across the cluster is used to help visualize the orbitals.

Moreover, the phosphine-ligated clusters with sigma aromaticity have a λ_max_ originating from the HOMO – 1 → LUMO transition. When an electron-withdrawing ligand like PMe_3_ is used, the λ_max_ originates from the HOMO → LUMO transition. In summary, the maximum absorption wavelength of these clusters is given by a combination of transitions between the frontier’s orbitals mentioned above. A detailed overview is provided for the excitation energies and oscillator strengths as Supporting Information in Data Set S1.

## Conclusions

We presented theoretical evidence to support the findings regarding highly stable phosphine-ligated Pt clusters containing two to five atoms. We found that the Pt_2_L_4_^2+^ (L = PH_3_, PMe_3_, PF_3_) cluster formed a covalent bond between the Pt atoms, whereas the neutral counterpart cluster was held together by an *n*_(Pt)_ → LV_(Pt)_ interaction. For clusters larger than three Pt atoms, neutral clusters were found to have higher stability than their charged counterparts. Because of the three-dimensional structure of these clusters, the number of electrons available for bonding, according to eq 1, agrees with the number of Pt atoms in the cluster (2e-per bond), as described by the NBO analysis. From a molecular orbital perspective, the high stability of the neutral clusters is due to the σ-aromaticity in both phosphine-ligated and trimethyl phosphine-ligated clusters. We conclude that the ligands influence the bonding of the core Pt atoms because of the steric hindrance or the type of atoms present. This statement is true regardless of the level of theory employed in our computations. In the neutral Pt_2_L_4_ cluster, the bonding interactions were strengthened using electron-withdrawing groups. In addition, using eq 1, we could predict the most stable clusters by calculating the number of electrons involved in the bonding. Finally, our time-dependent DFT results show that the wavelength of maximum absorption in clusters with sigma aromaticity is due to a transition from the HOMO – 1 to the LUMO. This finding is of great importance because ligand-induced sigma-aromaticity can influence the optical properties of Pt nanoclusters..

## Supporting Information Available

Electron density showing sigma aromaticity in phosphine and trimethylphosphine-ligated platinum nanoclusters containing 3–5 atoms and NBO charges for the Pt_2_L_2_ and Pt_2_L_2_^2+^ nanoclusters (L = PH_3_ and PMe_3_) (PDF)
