# Supplementary material for: Characterization of the Effects of Ligands on Bonding and σ-Aromaticity of Small Pt Nanoclusters
Source: J Phys Chem A. 2023 May 8;127(19):4237–44. doi: 10.1021/acs.jpca.2c08614 (PMC10201524; doi:10.1021/acs.jpca.2c08614)
Supplement: Supplementary file 1 — jp2c08614_si_001.pdf [file jp2c08614_si_001.pdf]

Characterization of the Effects of Ligand on Bonding and σ-Aromaticity of Small Pt Nanoclusters

Samantha Reid and Heriberto Hernández*

Department of Chemistry, Grinnell College, Grinnell, IA 50112

(a)

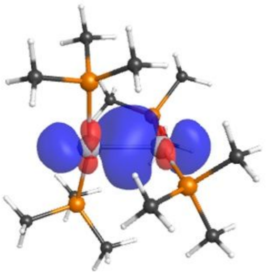

$$\boldsymbol{\psi^{+}_{Pt-Pt} = 0.7081sd^{1.30} + 0.7061sd^{1.26}}$$

(b)

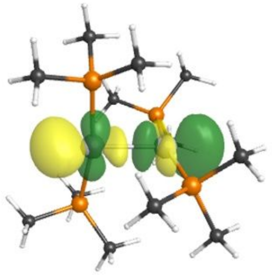

$$\boldsymbol{\psi^{-}_{Pt-Pt} = 0.7061sd^{1.30} - 0.7081sd^{1.26}}$$

Figure S1. $Pt_2(PMe_3)_4^{2+}$ nanocluster visual representation of (a) the Pt-Pt NBO sigma-type bonding orbital, $\sigma_{Pt\text{-}Pt}$, with a bond distance of 2.576 Å (PW91, 2.5550 Å) and (b) the Pt-Pt NBO sigma-type anti-bonding orbital. Underneath each figure is the wavefunction that describes the bonding (ψ^+) and anti-bonding (ψ^-) orbitals in the charged Pt (2) cluster.

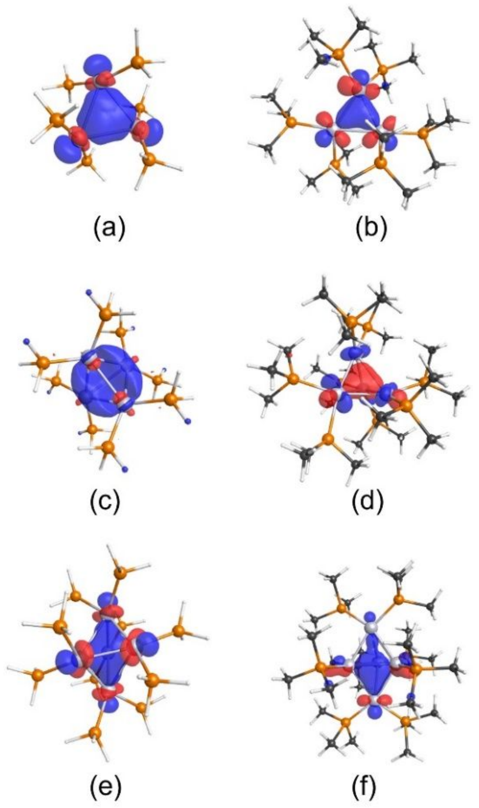

Figure S2. Sigma aromaticity was found in platinum nanoclusters containing 3 to 5 platinum atoms for both the phosphine (a, c and e) and trimethylphosphine-ligated platinum nanoclusters (b, d and f). Optimization was done using the B3LYP/def2TZV dkh2 level of theory.

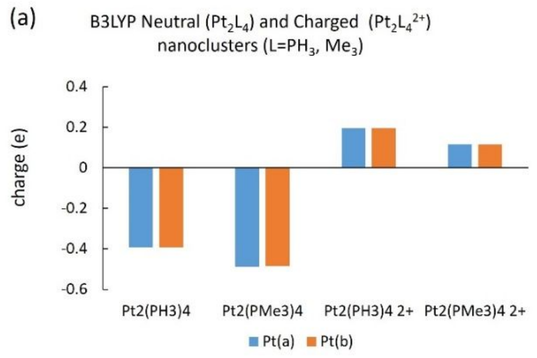

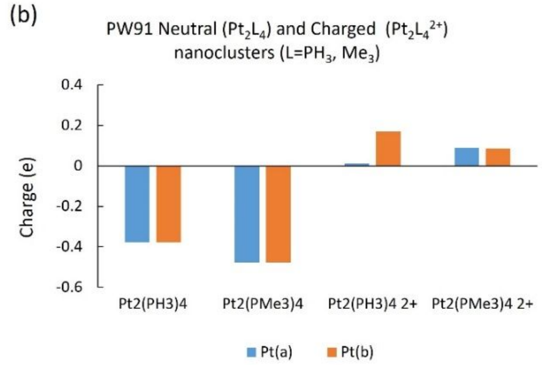

Figure S3. NBO charges for the Pt_2L_2 and $Pt_2L_2^{2+}$ nanoclusters (L=PH_3, PMe_3) (a) B3LYP/def2TZV dkh2 and (b) PW91/def2TZV dkh2 levels of theory.

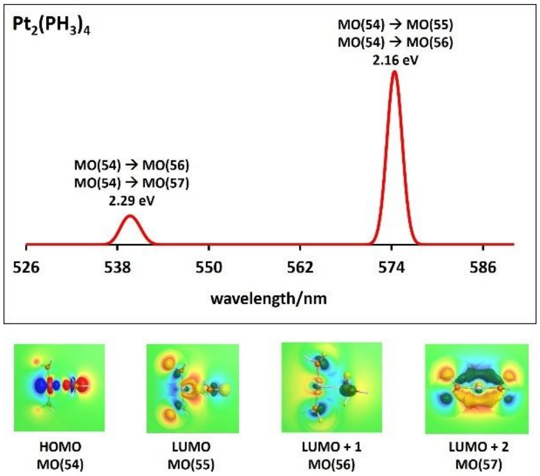

Figure S4. UV-Vis spectrum of the neutral $Pt_2(PH_3)_4$ cluster and its corresponding transitions resulting in the absorption spectrum. Underneath the spectrum are the plots of the molecular orbitals involved in the transitions. All the data was obtained using TD-DFT at the PW91/def2TZV dkh2 level of theory.

Data Set S1: Excitation energies and oscillator strengths.
TD-DFT
Level of Theory: PW91/def2TZV dkh2
Highlighted in blue is the wavelength of maximum absorption, λ_{max}.
Highlighted in red are the HOMO → LUMO transition.

Pt2(PH3)4

```
Excited state symmetry could not be determined.
 Excited State   1:      Singlet-?Sym    2.1585 eV 574.40 nm  f=0.0465  <S**2>=0.000
      51 -> 55        -0.13461
      54 -> 55         0.58505
      54 -> 56         0.36409
 This state for optimization and/or second-order correction.
 Total Energy, E(TD-HF/TD-KS) =  -1614.21840045
 Copying the excited state density for this state as the 1-particle RhoCI density.

 Excited state symmetry could not be determined.
 Excited State   2:      Singlet-?Sym    2.2940 eV  540.48 nm  f=0.0043  <S**2>=0.000
      54 -> 56         0.12168
      54 -> 57         0.69398

 Excited state symmetry could not be determined.
 Excited State   3:      Singlet-?Sym    2.2998 eV  539.10 nm  f=0.0054  <S**2>=0.000
      54 -> 58         0.70675
```

Pt2(PMe3)4

```
Excited state symmetry could not be determined.
 Excited State   1:      Singlet-?Sym    2.3707 eV  522.99 nm  f=0.0030  <S**2>=0.000
     102 ->104         0.70520
 This state for optimization and/or second-order correction.
 Total Energy, E(TD-HF/TD-KS) =  -2086.06387319
 Copying the excited state density for this state as the 1-particle RhoCI density.

 Excited state symmetry could not be determined.
 Excited State   2:      Singlet-?Sym    2.3928 eV  518.16 nm  f=0.0029  <S**2>=0.000
     102 ->105         0.70491

 Excited state symmetry could not be determined.
 Excited State   3:      Singlet-?Sym    2.4490 eV 506.27 nm  f=0.1308  <S**2>=0.000
      99 ->103        -0.18280
     100 ->105         0.14178
     101 ->104         0.16384
     102 ->103         0.64584
```

Pt3(PH3)3

```
Excited state symmetry could not be determined.
 Excited State   1:      Singlet-?Sym    1.9474 eV  636.66 nm  f=0.0012  <S**2>=0.000
      81 -> 82         0.70602
 This state for optimization and/or second-order correction.
 Total Energy, E(TD-HF/TD-KS) =  -2421.38960266
 Copying the excited state density for this state as the 1-particle RhoCI density.

 Excited state symmetry could not be determined.
 Excited State   2:      Singlet-?Sym    2.1721 eV  570.80 nm  f=0.0195  <S**2>=0.000
      78 -> 82         0.29248
      80 -> 82         0.57021
      81 -> 83        -0.27741

 Excited state symmetry could not be determined.
 Excited State   3:      Singlet-?Sym    2.1778 eV  569.30 nm  f=0.0199  <S**2>=0.000
      77 -> 82         0.30285
      79 -> 82         0.56254
      81 -> 84         0.28027
```

Pt3(PMe3)3

```
Excited state symmetry could not be determined.
 Excited State   1:      Singlet-?Sym    1.6065 eV  771.78 nm  f=0.0159  <S**2>=0.000
     149 ->154        -0.10695
     153 ->154         0.68999
 This state for optimization and/or second-order correction.
 Total Energy, E(TD-HF/TD-KS) =  -3129.15188290
 Copying the excited state density for this state as the 1-particle RhoCI density.

 Excited state symmetry could not be determined.
 Excited State   2:      Singlet-?Sym    1.9875 eV  623.83 nm  f=0.0087  <S**2>=0.000
     151 ->154         0.33138
     152 ->154         0.62209

 Excited state symmetry could not be determined.
 Excited State   3:      Singlet-?Sym    2.0456 eV  606.12 nm  f=0.0068  <S**2>=0.000
     150 ->154         0.31090
     151 ->154         0.55641
     152 ->154        -0.28173
```

Pt4(PH3)8

```
 Excited State   1:      Singlet-A      2.0306 eV  610.59 nm  f=0.0039  <S**2>=0.000
     108 ->109         0.69821
 This state for optimization and/or second-order correction.
 Total Energy, E(TD-HF/TD-KS) =  -3228.60876685
 Copying the excited state density for this state as the 1-particle RhoCI density.

 Excited State   2:      Singlet-A      2.0377 eV  608.46 nm  f=0.0048  <S**2>=0.000
     107 ->109         0.69834

 Excited State   3:      Singlet-A      2.4318 eV  509.85 nm  f=0.0000  <S**2>=0.000
     106 ->109         0.70682
```

Pt4(PMe3)8

```
 Excited state symmetry could not be determined.
 Excited State   1:      Singlet-?Sym    2.0085 eV  617.30 nm  f=0.0070  <S**2>=0.000
     204 ->205         0.69179
 This state for optimization and/or second-order correction.
 Total Energy, E(TD-HF/TD-KS) =  -4172.26189941
 Copying the excited state density for this state as the 1-particle RhoCI density.

 Excited state symmetry could not be determined.
 Excited State   2:      Singlet-?Sym    2.0308 eV  610.52 nm  f=0.0074  <S**2>=0.000
     203 ->205         0.68964

 Excited state symmetry could not be determined.
 Excited State   3:      Singlet-?Sym    2.3386 eV  530.18 nm  f=0.0174  <S**2>=0.000
     204 ->206         0.68661
```

Pt5(PH3)8

```
 Excited state symmetry could not be determined.
 Excited State   1:      Singlet-?Sym    1.5193 eV  816.05 nm  f=0.0001  <S**2>=0.000
     115 ->119        -0.14576
     117 ->118         0.69141
 This state for optimization and/or second-order correction.
 Total Energy, E(TD-HF/TD-KS) =  -3348.14406794
 Copying the excited state density for this state as the 1-particle RhoCI density.

 Excited state symmetry could not be determined.
 Excited State   2:      Singlet-?Sym    1.6562 eV  748.60 nm  f=0.0011  <S**2>=0.000
     116 ->118         0.70156
 Excited state symmetry could not be determined.
 Excited State   3:      Singlet-?Sym    1.7417 eV  711.85 nm  f=0.0007  <S**2>=0.000
     115 ->118        -0.35303
     117 ->119         0.61143
```

Pt5(PMe3)8

```
 Excited state symmetry could not be determined.
 Excited State   1:      Singlet-?Sym    1.5905 eV  779.54 nm  f=0.0011  <S**2>=0.000
     213 ->214         0.68775
     213 ->215        -0.14307
 This state for optimization and/or second-order correction.
 Total Energy, E(TD-HF/TD-KS) =  -4291.81489781
 Copying the excited state density for this state as the 1-particle RhoCI density.

 Excited state symmetry could not be determined.
 Excited State   2:      Singlet-?Sym    1.6234 eV  763.73 nm  f=0.0003  <S**2>=0.000
     211 ->214        -0.11264
     211 ->215        -0.14587
     212 ->214         0.64475
     212 ->215        -0.21818

 Excited state symmetry could not be determined.
 Excited State   3:      Singlet-?Sym    1.6504 eV  751.25 nm  f=0.0003  <S**2>=0.000
     213 ->214         0.14236
     213 ->215         0.69024
```

Pt5(PH3)10

```
 Excited state symmetry could not be determined.
 Excited State   1:      Singlet-?Sym    1.3827 eV  896.69 nm  f=0.0319  <S**2>=0.000
     135 ->136         0.69340
 This state for optimization and/or second-order correction.
 Total Energy, E(TD-HF/TD-KS) =  -4035.78969562
 Copying the excited state density for this state as the 1-particle RhoCI density.

 Excited state symmetry could not be determined.
 Excited State   2:      Singlet-?Sym    1.6760 eV  739.74 nm  f=0.0081  <S**2>=0.000
     133 ->136         0.37046
     134 ->136         0.58915

 Excited state symmetry could not be determined.
 Excited State   3:      Singlet-?Sym    1.7184 eV  721.50 nm  f=0.0043  <S**2>=0.000
     133 ->136         0.58913
     134 ->136        -0.37750
```

Pt5(PMe3)10

```
Excited State   1:      Singlet-A      1.5418 eV  804.15 nm  f=0.0133  <S**2>=0.000
    254 -> 256        0.16242
    255 -> 256        0.68040
This state for optimization and/or second-order correction.
Total Energy, E(TD-HF/TD-KS) =  -5215.30956206
Copying the excited state density for this state as the 1-particle RhoCI density.

Excited State   2:      Singlet-A      1.7888 eV  693.12 nm  f=0.0279  <S**2>=0.000
    254 -> 256        0.68014
    255 -> 256       -0.14787

Excited State   3:      Singlet-A      1.8408 eV  673.54 nm  f=0.0176  <S**2>=0.000
    253 -> 256        0.68758
```
